# Human parasitic infections of the class Adenophorea: global epidemiology, pathogenesis, prevention and control

**DOI:** 10.1186/s40249-024-01216-1

**Published:** 2024-06-20

**Authors:** Jitrawadee Intirach, Chang Shu, Xin Lv, Suzhen Gao, Nataya Sutthanont, Tao Chen, Zhiyue Lv

**Affiliations:** 1 Hainan General Hospital, Hainan Affiliated Hospital of Hainan Medical University, Haikou, Hainan 570100 China; 2 School of Basic Medical Sciences and Life Sciences, Hainan Medical University, Haikou, Hainan 571199 China; 3 School of Public Health, Hainan Medical University, Haikou, Hainan 571199 China; 4 Department of Medical Entomology, Faculty of Tropical Medicine, Mahidol University, Bangkok, 10400 Thailand; 5 Hainan Provincial Bureau of Disease Prevention and Control, Haikou, 570100 China; 6 Provincial Engineering Technology Research Center for Biological Vector Control, Guangzhou, Guangdong 510080 China; 7 Key Laboratory of Tropical Disease Control (Sun Yat-Sen University), Ministry of Education, Guangzhou, Guangdong 510080 China

**Keywords:** Adenophorea, Morphological, Life cycle, Global epidemiology, Pathology

## Abstract

**Background:**

Human parasitic infections caused by Adenophorean nematodes encompass a range of diseases, including dioctophymiasis, trichuriasis, capillariasis, trichinellosis, and myositis. These infection can result in adverse impacts on human health and cause societal and economic concerns in tropical and subtropical regions.

**Methods:**

This review conducted searches in PubMed, Embase and Google Scholar for relevant studies that published in established databases up to April 26, 2024. Studies that focused on the common morphology, life cycle, disease distribution, clinical manifestations, and prevention and control strategies for Adenophorean parasitic diseases in humans were included.

**Results:**

Adenophorean nematodes exhibit shared morphological characteristics with a four-layered cuticle; uninucleate epidermal cells; pseudocoelom with six or more coelomocytes; generally three caudal glands; five esophageal glands; two testes in males with median-ventral supplementary glands in a single row; tail in males rarely possessing caudal alae; amphids always postlabial; presence of cephalic sensory organs; absence of phasmids; and a secretory-excretory system consisting of a single ventral gland cell, usually with a non-cuticularized terminal duct. Humans play two important roles in the life cycle of the nematode class, Adenophorea: 1) as a definitive host infected by ingesting undercooked paratenic hosts, embryonated eggs, infective larvae in fish tissue and meat contaminated with encysted or non-encysted larvae, and 2) as an accidental host infected by ingesting parasitic eggs in undercooked meat. Many organs are targeted by the Adenophorean nematode in humans such as the intestines, lungs, liver, kidneys, lymphatic circulation and blood vessels, resulting in gastrointestinal problems, excessive immunological responses, cell disruption, and even death. Most of these infections have significant incidence rates in the developing countries of Africa, Asia and Latin America; however, some parasitic diseases have restricted dissemination in outbreaks. To prevent these diseases, interventions together with education, sanitation, hygiene and animal control measures have been introduced in order to reduce and control parasite populations.

**Conclusions:**

The common morphology, life cycle, global epidemiology and pathology of human Adenophorean nematode-borne parasitic diseases were highlighted, as well as their prevention and control. The findings of this review will contribute to improvement of monitoring and predicting human-parasitic infections, understanding the relationship between animals, humans and parasites, and preventing and controlling parasitic diseases.

**Graphical Abstract:**

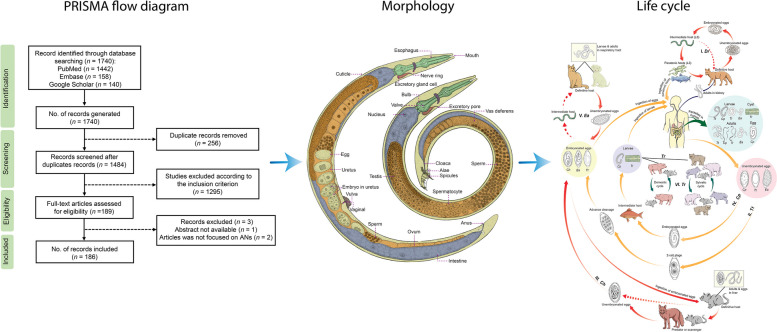

**Supplementary Information:**

The online version contains supplementary material available at 10.1186/s40249-024-01216-1.

## Background

Adenophorea Chitwood, 1958 (also known as Enoplea Inglis, 1983 or Aphasmidia) is a class of phylum Nematoda (roundworms), initially classified into two classes by Chitwood [1]: Phasmidia and Aphasmidia, based on the presence and absence of phasmids, respectively. However, Chitwood [2] later proposed replacing these terms with Secernentea (= secretors, referring to the presence of an excretory system with lateral canals) and Adenophorea (= gland bearers, referring to the presence of caudal glands) as modifications of the term coined by von Linstow [3]. In some classifications, Adenophorea would be grouped with Enoplea, and Secernentea with Chromadorea Inglis, 1983, based on small subunit (SSU) rDNA sequences [4, 5]. Most Adenophoreans are free-living, aquatic, and microbotrophic nematodes. They also are species that cause parasitic disease in invertebrates, vertebrates and plants [6]. The Adenophorea class has three major genera; *Trichinella* Railliet, 1895, *Capillaria* Zeder, 1800 and *Trichuris* Roederer, 1761, all of which are infectious in humans and remain as important public health issues worldwide, particularly in developing countries [7]. *Trichinella*, *Trichuris* and *Capillaria *sensu lato belong to the superfamily, Trichinelloidea Ward, 1907, and their stichosome esophagus has a unique structure [8] that resembles a capillary tube equating to a stack of gland cells, known as stichocytes, which are arranged similarly to a stack of donuts. Based on complete mtDNA coding genes, the order Trichocephalida Spasski, 1954 is segregated into three well-defined clades, representing the families Trichinellidae Ward, 1907, Trichuridae Ransom, 1911 and Capillariidae Railliet, 1915, which include *Trichinella*, *Trichuris* and *Capillaria*, respectively [9].

**Fig. 1:**
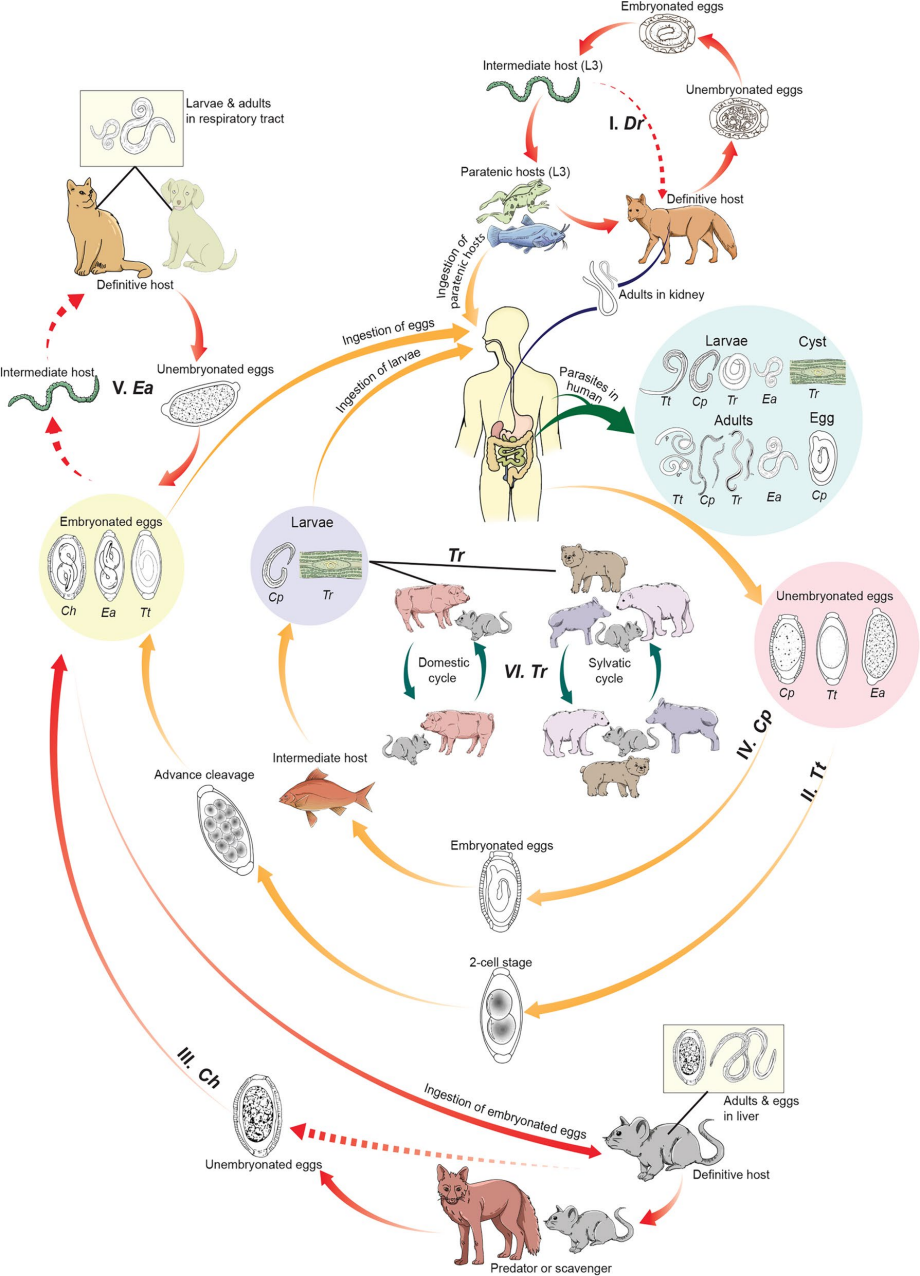
Schematic illustration of the life cycles of six parasites (redrawn from Centers for Disease Control and Prevention [28–32], including *E. aerophilus*,* C. hepaticum*, *C. philippinensis*, *D. renale*, *Trichinella* spp. and *T. trichuira*. *Ea* *Eucoleus aerophilus*; *Ch* *Calodium hepaticum*; *Cp* *Capillaria philippinensis*; *Dr* *Dioctophyme renale*;* L3* third-stage larvae; Tr *Trichinella* spp.; *Tt* *Trichuris trichuira*

*Trichinella* spp. has been identified as the causative agent for human trichinellosis in many regions globally [10]. Over the past two decades, reports on *Trichinella* spp. infections in animals and humans have emerged from 95 (48.5%) countries worldwide. These cases encompass both the wild cycle, documented in 75 countries (38.3%), and domestic cycle, noted in 32 countries (16.3%), as well as human infections in 47 countries (23.9%). Nevertheless, there remains a scarcity of recent epidemiological data in numerous countries, with some available information dating back to the previous century [10]. Trichinellosis had an estimated annual incidence rate of 469.2‒985.3 cases per billion individuals per year and a global mortality rate of 0.3‒0.8 per billion individuals per year [11]. While the *Capillaria* species primarily infest various vertebrate animals, only three of them are known to infect humans: *Calodium hepaticum* (syn. *Capillaria hepatica* Bancroft, 1893), *Eucoleus aerophilus* Dujardin, 1845 (*Capillaria aerophila* Creplin, 1839) and *Capillaria philippinensis* Velasquez, Chitwood and Salazar, 1968 [12]. Intestinal capillariasis, primarily caused by *C. philippinensis,* is the most significant disease, which exhibits a higher prevalence in humans when compared to other *Capillaria* species. Furthermore, human trichuriasis is an intestinal helminthic infection, widespread globally, and attributed to the whipworm parasite *Trichuris trichiur*a Linnaeus, 1771 [13, 14]. It is estimated that approximately 25% of the global population is infected with this parasite. *Trichuris trichiura* is found more commonly in specific regions, particularly in tropical and subtropical areas like East Asia, China, sub-Saharan Africa, and the Americas [15]. Roughly 1.049 billion individuals are affected by *T. trichiura*, encompassing 114 and 233 million preschool- and school-age children, respectively. The occurrence of *T. trichiura* is elevated and can be as high as 95% among children in various regions worldwide. These areas often experience issues like protein energy malnutrition and anemia, together with limited access to healthcare and educational resources [16]. *Trichuris* species that have been identified in various mammals include *Trichuris vulpis* Froelich, 1789 (found in canines), *Trichuris suis* Schrank, 1788 (in swine), *Trichuris skrjabini* Baskakov, 1924 (in goats), *Trichuris ovis* Abildgaard, 1795 (in sheep) and *Trichuris muris* Schrank, 1788 (in mice). Among these, only *T. suis* [17] and *T. vulpis* [18, 19] are capable of causing persistent active infections in humans. In addition, Mohd-Shaharuddin [20] reported use of molecular methods to test for parasite eggs, and the presence of *T. trichiura* in dog feces was revealed. Interestingly, *T. vulpis* also has been identified in human feces (1.3%). These findings suggest that cross-infection between humans and animals in sympatric areas may contribute to infections in both hosts. *Dioctophyme renale* Goeze, 1782, and *Haycocknema perplexum* Spratt, Beveridge, Andrews & Dennett, 1999 are two species in the class that can infect humans, but they have not been reported widely [21, 22]. *Dioctophyme renale* can be found across the globe, but is seldom responsible for human infections [23]. So far, reports of human cases have only emerged from 10 countries. In China, a total of 21 human cases have been recorded since the initial report of dioctophymosis in humans in 1981, spanning at least 14 provinces and municipalities [22]. Furthermore, *H. perplexum* is a parasite that has been associated with infrequent cases of life-threatening myositis in humans. This condition has only been documented in nine patients in Australia from 1998 to 2016 [24]. Currently, its presence is extending beyond its original endemic regions in Australia, encompassing areas like tropical north Queensland and Tasmania [21, 24]. Millions of people aross approximately 145 countries have been affected by outbreaks of Adenophorean nematodes (ANs). These outbreaks occur when humans ingest food or water contaminated with the eggs or encysted larvae of these worms (Fig. 1). Among this class of parasitic nematodes, *T. trichiura* is the species known to parasitize humans [25], while others are predominantly zoonotic [26, 27]. Thus, the main focus of prevention and control has been on animal control and hygiene measures.

Previous studies have documented the prevalence, pathogenesis, and distribution of ANs [33]. However, due to extensive information needing collection, new data are required to update understanding of them. This review study focused on diverse parasite species and the global distribution of parasitic diseases involving ANs in humans. Moreover, deeper understanding of the basic biology of nematodes in the Adenophorea class and their reservoir is necessary in order to explain the expanding geographical distribution of these diseases [34, 35]. This review article aimed to provide a comprehensive overview of human parasitic infections involving ANs. To achieve this, a systematic review of existing literature was undertaken. The search focused on scholarly articles and publications that investigated the prevalence, pathogenesis, and clinical aspects of AN infections in humans. Additionally, studies exploring the common morphological features, life cycle characteristics of these parasites, as well as strategies for preventing and controlling AN-related diseases were included. Through the examination of these diverse aspects, this review aimed to enhance current knowledge and understanding of AN-related human parasitic infections.

## Methods

### Information sources and search strategy

This scoping review followed the recommendations outlined in the Preferred Reporting Items for Systematic Reviews, and Meta-analyses Extension for Scoping Reviews (PRISMA-ScR) guidelines [36]. A combination of search terms was analyzed across various data sources, including PubMed, Embase, and Google Scholar, in order to conduct the scoping review from inception of the databases up until April 26, 2024. These sources were searched systematically for relevant books, articles, and other material. The scope of this review was centered around the Adenophorea class of nematodes, which has the potential to impact human health. The search strategy comprised a combination of keywords found in titles or abstracts, which were formulated as follows: Adenophorea (every genus such as *Trichinella*, *Trichuris*, *Capillaria*, etc.) AND (human parasites OR parasite infections OR parasite prevalence) AND (nematode morphology OR nematode life cycle OR global epidemiology of nematodes OR nematode pathology in humans OR prevention and control of nematodes) (see Additional file 1: Table S1 for detailed strategies).

Duplicated records were identified and removed using EndNote software X9 (Clarivate, Philadelphia, PA, USA). All of the papers underwent initial screening based on their titles and abstracts to identify the most relevant studies. If necessary, the morphological characteristics, life cycle, epidemiology, global distribution, pathogenesis, and prevention and control strategies relating to Adenophorea parasitic diseases were summarized.

### Inclusion and exclusion criteria

The entire texts of these studies were examined to determine their suitability for inclusion in the review. The criteria for inclusion were as follows: 1) relevance to the Adenophorea class of nematodes, 2) explanation of the common morphology of the Adenophorea class of nematodes, 3) information related to the life cycle, epidemiology, pathogenicity, and prevention and control of human parasitic diseases caused by the Adenophorea class of nematodes, 4) no specified starting date for the databases, with a cutoff date of April 26, 2024, 5) cases or outbreaks occurring worldwide, and 6) publications written in English. In addition, studies presenting findings from the same source, conference abstracts, comments, resources without references, or studies that did not meet the relevant criteria were excluded.

### Quality assessment of included literature

Critical appraisal was conducted by four reviewers (JI, CS, XL, SZG), who utilized the "Crowe Critical Appraisal Tool" (CCAT) to assess the quality of each study [37]. The CCAT, known for its reliability and validity in evaluating studies with diverse designs and implementation approaches, helped in reducing rater bias [37, 38]. To mitigate potential bias further, individual assessments were cross-checked, and any discrepancies were resolved by a fifth reviewer (NS). By following the established guidelines, the study team decided to categorize articles based on their CCAT scores, which used a six-point scale (ranging from 0 to 5 for each category), with a possible total score of 40.

### Evidence extraction and analysis

Information was extracted from the studies included, based on the following categories: 1) morphology classification of Adenophorea, 2) life cycle of human Adenophorea parasitic diseases, 3) epidemiological trends and pathogenicity of human Adenophorea parasitic diseases, and 4) prevention and control strategies for human Adenophorea parasitic diseases. Five authors (JI, CS, XL, SZG and NS) independently evaluated the qualifications of the studies, compiled relevant information, and cross-validated the findings. Any disagreements were resolved through consensus among the authors.

### Gap analysis

A gap analysis was conducted to identify areas that lacked research or had insufficient evidence. This analysis aimed to highlight knowledge gaps in current understanding of the Adenophorea class of nematodes and their impact on human health.

## Results

**Fig. 2:**
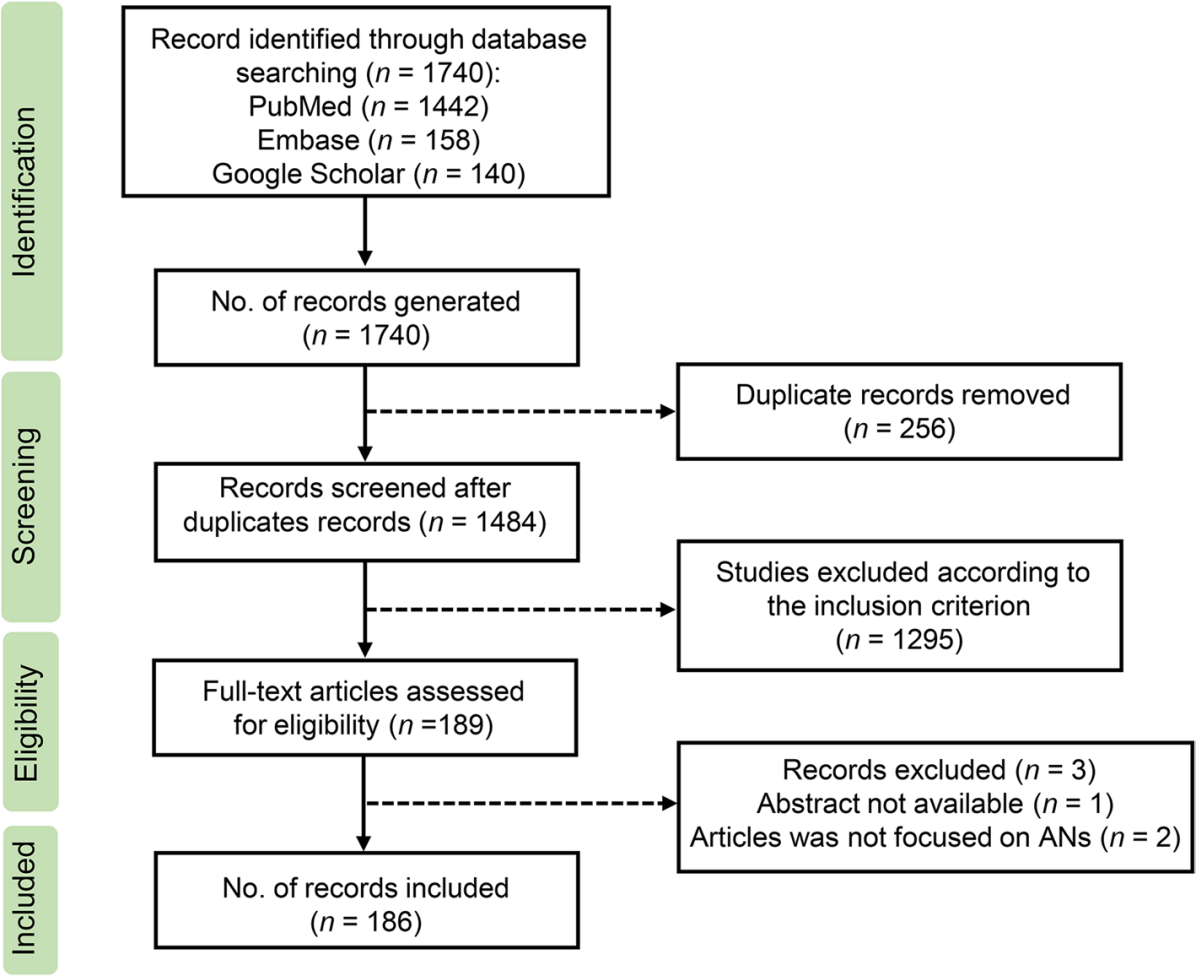
PRISMA flow diagram of the scoping review process for the Adenophorean class of nematodes

A total of 1740 records were retrieved initially for this review. After removing 256 duplicate records and excluding 1295 that did not meet the specified criteria, 186 were included in the analysis. Figure 2 presents a flowchart illustrating the selection process.

### Common morphological characteristics of Adenophorea

**Fig. 3:**
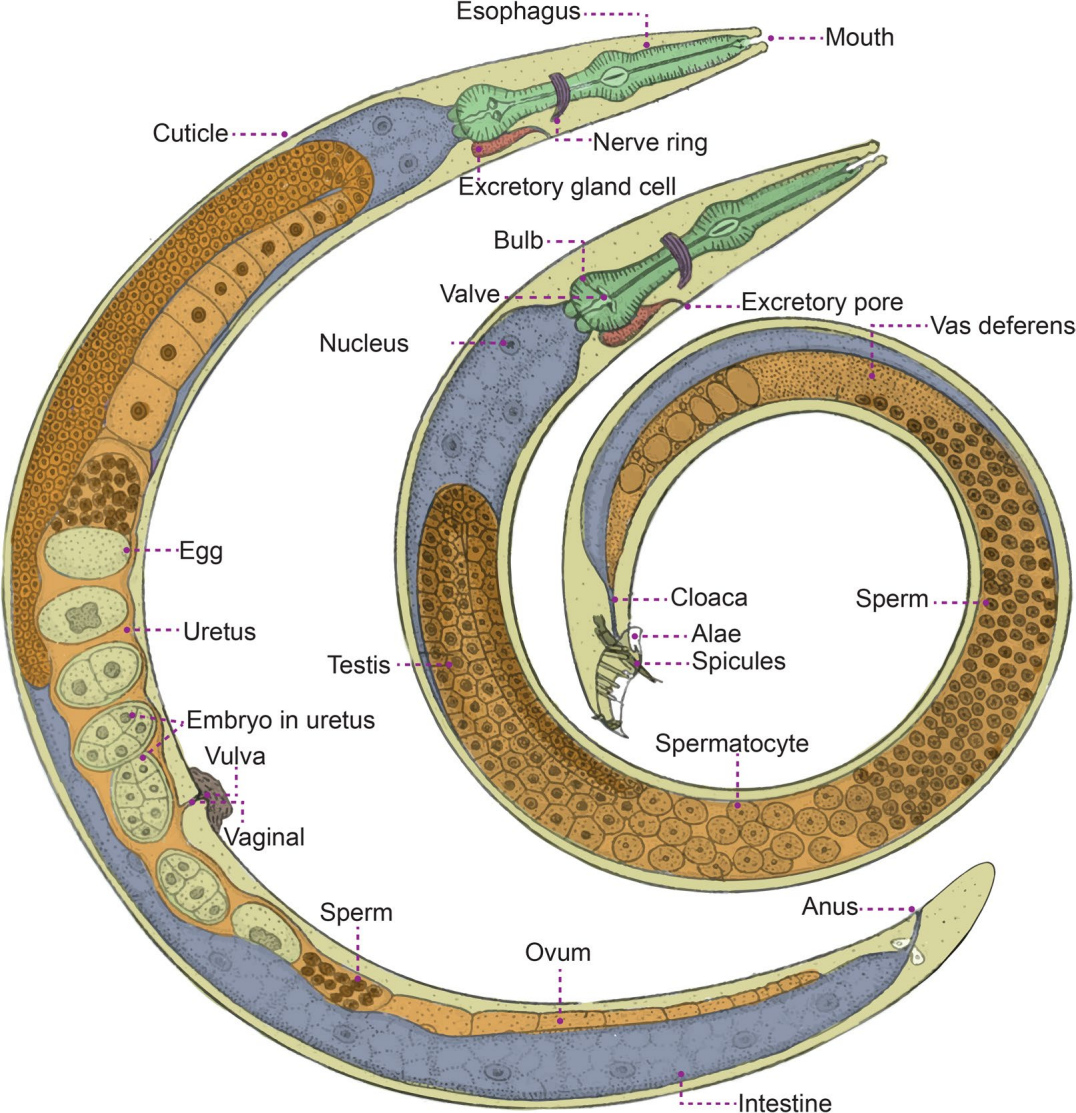
The common morphological characteristics of the Adenophorea class, phylum Nematoda (redrawn from Chitwood & Chitwood, 1950 [51]). Female (left) and male (right)

Adenophorea is characterized by specific morphological features encompassing the cuticle, epidermis, pseudocoelom, and the digestive, reproductive, sensory/nervous, and secretory-excretory systems [2, 6, 39–46]. Figure 3 details the morphological characteristics of the Adenophorea class, phylum Nematoda. The cuticle serves as a flexible exoskeleton, and exhibits invaginations at various points, including the mouth, amphids, aphasmids, and reproductive and excretory openings [39]. While generally smooth, the cuticle may display transverse or longitudinal striations that contain four layers of cuticle [6, 41]. Epidermal glands are often found beneath the cuticle, consisting of both unicellular epidermal glands and nonglandular cells [39]. Adenophoreans are non-segmented pseudocoelomates with a three-layered body, housing a pseudocoelom lined between the mesoderm and endoderm [39, 44], which contain six or more coelomocytes [46]. The digestive system typically lacks a rectal gland, but possesses three caudal and five esophageal glands [2, 6, 43]. The anterior portion of the specialized esophagus is a thin-walled, muscular tube in Trichinellida and Mermithida, while its posterior portion consists of a very thin tube surrounded by a column of single glandular cells called stichocytes. This entire structure is referred to as a stichosome. Stichocytes meet with the esophageal lumen via small ducts [47]. The stichosome may be homologous to the esophageal glands found in other nematodes, possibly arising from multiplication of the number of glands [48]. Eggs in the order, Trochocephalida (including *Trichuris* and *Capillaria* s.l.), typically have bipolar plugs (opercula), except in *Trichinella* spp. [49]. Similarly, eggs in the order, Dioctophymatida (*Dioctophyme*), are sculptured deeply or pitted and contain clear bipolar plugs [50]. The male reproductive system includes two testes, male tail with barely caudal alae (or bursae), and supplement glands that are median-ventral in a single row [6, 40, 43]. Adenophoreans feature paired lateral chemosensory organs known as amphids and lack deirids and phasmids [6, 40, 42]. Somatic and cephalic sensilla are distributed throughout the body, with cephalic sensilla positioned around the head [6, 42, 46]. The secretory-excretory system consists of a single ventral gland cell with a noncuticularized terminal duct lacking collecting tubules [6, 40, 45].

### Life cycles of human Adenophorea parasitic diseases

Human parasitic diseases that are caused by ANs can be classified into seven groups, based on their genus and species (Fig. 1).

#### Group I

Humans serve as definitive hosts of *D. renale* following the consumption of undercooked paratenic hosts, such as fish or frogs [52]. Eggs of the parasite are excreted in urine, hatch into first-stage larvae (L_1_) in water, and are then ingested by oligochaete worms (intermediate hosts). These larvae develop to the third stage (L_3_) and encyst within paratenic hosts, which can subsequently infect carnivorous mammals and humans through their consumption. Humans can become infected by consuming either the paratenic or intermediate host [53, 54].

#### Group II

Humans are the definitive hosts of *T. trichuira*, which has no intermediate host in its life cycle. It releases unembryonated eggs into the soil together with feces, from which a 2-cell, advanced cleavage develops into embryonated eggs [55]. Humans are infected by ingesting the embryonated eggs in soil-contaminated food. Upon ingestion, the eggs hatch in the small intestine of the human, and the larvae mature in the colon [53].

#### Group III

Humans are the second accidental host of *C. hepaticum*. They are infected through the ingestion of parasitic eggs present in fecally contaminated water, food, or soil. *C. hepaticum* has a direct life cycle without an intermediate host [56, 57]. Eggs hatch in the intestine of rodents, thus releasing L_1_ larvae that develop and lay eggs in their livers [53]. The eggs are retained in the liver until the animal dies, and then released into the environment, as predators or scavengers (first accidental host) ingest the definitive host, and the cycle continues repeatedly [58]. In humans, the larvae hatch in the intestine, and juveniles migrate to the liver, mature, mate, and lay eggs, causing granuloma formation, liver necrosis [58, 59] and lesions [60].

#### Group IV

Humans are the definitive hosts for *C. philippinensis*. They are infected by consuming larvae in fish tissue. Unembryonated eggs are discharged in human feces and develop in water. When fish ingest these eggs, larvae develop in their tissues, and humans consume the infected raw or undercooked fish. Adult worms live in the small intestine, and females lay unembryonated and shell-less embryonated eggs. Released larvae can re-invade the small intestine in an autoinfection cycle [61, 62].

#### Group V

Humans may act as definitive hosts of *E. aerophilus*. They are infected by ingesting embryonated eggs from soil. This worm has a direct life cycle. The larvae hatch in the small intestine, then perforate the mucosa, migrate to the lungs, penetrate the alveoli, relocate to the air passages as they develop, and eventually reach maturity to live in the respiratory epithelium. Females lay eggs, which are excreted into the soil to mature. *E. aerophilus* may also have an indirect life cycle involving earthworms as intermediate hosts, which become infected after consumption by a mammalian host [63].

#### Group VI

Humans are the definitive host of *Trichinella* spp. They become infected by swallowing meat contaminated by encysted larvae (except for *T. pseudospiralis* and *T. papuae*, which do not encyst). The larvae develop into adult worms in the small intestine, in which they release larvae that migrate to muscle cells. Adults remain in the intestine until they die, and are then excreted [33]. Infection sources include domestic and wild animals [64].

#### Group VII

*H. perplexum* is a small muspiceoid nematode found within human skeletal muscle cells [65]. Its life cycle is still unknown. Potential sources of human infection encompass vertebrates, invertebrates, plants, soil, and water [5, 66]. Speculation suggests that it may rely on an intermediary arthropod host, with humans serving as incidental hosts; or that humans contract the infection through consuming of undercooked bush-meat from infected native fauna [21].

In summary, humans are accidental hosts of *C. hepaticum* but serve as definitive hosts of *D. renale*, *T. trichuira*, *C. philippinensis*, *E. aerophilus*, and *Trichinella*. It remains unclear whether humans are the host for *H. perplexum*. These parasites undergo various developmental stages within humans and other hosts, demonstrating the complexity of their life cycles. Similarities in these parasites include humans often acting as definitive hosts and common infection routes through ingestion of contaminated food or water. However, there are differences in life cycle complexity, with some parasites having direct cycles without intermediate hosts (*T. trichiura*, *C. hepaticum*), while others involve multiple hosts (*D. renale*, *C. philippinensis*, *E. aerophilus*). Additionally, infection mechanisms and adult worm locations vary, affecting organs such as the kidneys, colon, liver, small intestines, lungs, and muscle cells.

### Epidemiology and pathogenesis of human Adenophorea parasitic diseases

The Adenophorea class of nematodes remains a public health concern of importance regarding humans. The distribution of human infection caused by 15 parasite species and 5 families in this class are described in Table 1.

**Table 1:** Distribution of Adenophorean nematodes that can transmit parasitic diseases, human infection cases, host range and source of infection in humans

**Categories**	**Distribution**	**Distribution of human cases**	**Host range or host infection**	**Source of infection in humans**	**References**
Capillaridae Railliet, 1915					
*Eucoleus aerophilus* Dujardin, 1845 (syn. *Capillaria aerophila* Creplin, 1839)	Worldwide		Wild carnivores, dogs, cats, humans	Ingestion of embryonated eggs	[67]
		France, Iran, Morocco, Russia, Serbia, Ukraine	Carnivorous mammals	Cats	[68]
*Calodium hepaticum* (Bancroft, 1893) Moravec, 1982 (syn. *Capillaria hepatica* Bancroft, 1893)	Worldwide		Murid rodents and various other mammals	Consumption of unembryonated eggs in soil or infected game	[69]
		Argentina, France	Human, rodent, camelid	ND	[70]
		Brazil, Canada, Czechoslovakia, England, Germany, Greece, Italy, Mexico, New Zealand, Nigeria, India, Ivory Coast, Japan, Republic of Korea, South Africa, Switzerland, Thailand, Turkey, USA, Yugoslavia	Marsupials, carnivores, hominids and other mammals	The consumption of infected game or from soil	[58]
		China	Human	–	[60]
		Israel	Human	–	[59]
		Iran	Human	–	[57]
*C. philippinensis* Velasquez, Chitwood and Salazar, 1968	The Philippines, Thailand, Japan, Iran, Egypt, China		Human	Raw fish	[71]
		China	Human	Fish	[72]
		Colombia	Human	Fish	[73]
		Egypt	Human	Fish	[74–76]
		India	Human	Fish	[77]
		Indonesia	Human	–	[78]
		Japan	Human	Fish	[79]
		Republic of Korea	Human	Fish	[80, 81]
		Lao People's Democratic Republic	Human	Fish	[82]
		The Philippines	Human	Fish	[62, 83, 84]
		China (Taiwan Province)	Human	Fish	[85, 86]
		Thailand	Human	Fish	[87–89]
Dioctophymatidae Castellani & Chalmers, 1910					
*Dioctophyme renale* Goeze, 1782	Worldwide		Mustelids, wild carnivores, domestic animals	Ingestion of raw or undercooked fish or frogs	[22, 54]
		Australia, China, Greece, India, Iran, Indonesia, Japan, Thailand, the United States, and Yugoslavia	Mustelids, wild carnivores, domestic animals	Fish or frogs	[22, 54]
Robertdollfusidae Chabaud & Campana, 1950					
*Haycocknema perplexum* Spratt, Beveridge, Andrews & Dennett, 1999	ND	Queensland and Tasmania in Australia	ND	ND	[21, 90]
Trichinellidae Ward, 1907					
*Trichinella britovi* (T3) Pozio, la-Rosa, Murrell & Lichtenfels, 1992	Temperate areas of Europe and Asia, Northern and Western Africa		Sylvatic mammals and seldomly domestic pigs	Wild boars, domestic pigs horses, foxes, jackals	[33]
		Algeria	Human	Golden jackal (*Canis aureus*)	[91]
		Bulgaria	Human	Wild boar and backyard pig	[92]
			Human	Domestic pig	[93]
		France	Human	Wild boar	[94]
		Greece	Human	Wild boar meat products	[95]
		Italy	Human	Wild boar meat products	[96]
			Human	Wild boar and pig	[97]
		Serbia	Human	Wild boars	[98]
		Slovakia	Human	Red foxes and wild boars	[99]
		Spain	Human	Wild boar	[100]
			Human	Wild boar	[101]
		Sweden	Human	Wild boar	[101]
		Turkey	Human	Raw meat balls of beef-mixed pork	[102]
*T. murrelli* (T5) Pozio & La Rosa, 2000	United States and southern Canada		Sylvatic carnivores	Bears, horses	[33]
		France	Human	Horse	[103]
*T. nativa* (T2) Britov & Boev, 1972	Arctic and subarctic areas of America, Asia, Europe		Sylvatic carnivores	Bears, walruses	[33]
		Canada (northern Ontario)	Human	Black bear meat	[104]
		Canada (northern Saskatchewan)	Human	Bear meat	[105]
		China	Human	Dog	[106]
		United States	Human	Cougar jerky	[107]
*T. nelsoni* (T7) Britov and Boev, 1972	Eastern-southern Africa		Sylvatic mammals	Warthogs, bush pigs	[33]
		Ethiopia, Kenya, Tanzania and Senegal	Human	–	[108]
*T. papuae* (T10) Pozio et al., 1999	Papua New Guinea, Thailand		Wild pigs, saltwater crocodiles	Wild pigs	[33]
		Cambodia	Human	Wild pig	[109]
		Malaysia	Human	Wild boar	[110]
		Thailand	Human	Wild pig	[111]
				Wild boar	[112]
*T. pseudospiralis* (T4) Garkavi, 1972	Cosmopolitan		Sylvatic mammals and birds, domestic pigs	Domestic and wild pigs	[33]
		France	Human	Wild boar	[113]
		Italy	Human	The ‘beef’ steak tartare	[114]
		Russia (Kamchatka)	Human	–	[115]
		Tasmania	Human	–	[116]
		Thailand	Human	Wild pig	[117]
*T. spiralis* (T1) Owen, 1835	Cosmopolitan		Domestic and sylvatic mammals	Domestic and sylvatic swine horses	[33]
		Austria	Human	Pork belly	[118]
		Bulgaria	Human	Backyard pig	[92]
				Domestic pigs	[93]
				Insufficiently cooked meat	[119]
		Canada	Human	Sheep and pigs	[120]
		Chile	Human	Wild boar	[121]
		Croatia	Human	Smoked sausages	[122]
				–	[123]
				Insufficiently cooked meat	[119]
		Egypt	Human	Pigs	[124]
		Estonia	Human	–	[125]
		Ethiopia	Human	Raw pork meat	[126]
		France Serbia	Human	Backyard pigs	[127]
				Insufficiently cooked meat	[119]
		Germany, Latvia, Lithuania, Russia, Spain	Human	Insufficiently cooked meat	[119]
		China (Hong Kong Special Administrative Region)	Human	Inadequately cooked pork	[128]
		Hungary	Human	Backyard pigs and wild boars	[129]
		India	Human	Pork	[130]
		Indonesia	Human	–	[131]
		Italy	Human	Pigs, wild boars and horses	[97]
				Insufficiently cooked meat	[119]
		Republic of Korea	Human	Wild boar	[132]
				Badger	[133]
		Lao People's Democratic Republic	Human	Uncooked or fermented pork	[134]
		Lebanon	Human	Pork	[135]
		Mexico	Human	–	[136]
		New Zealand	Human	Pigs	[137]
		Poland	Human	Sausage made of wild boar	[138]
				Insufficiently cooked meat	[119]
		Romania	Human	Pig meat and products (including wild boar)	[123]
				Insufficiently cooked meat	[119]
		Slovakia	Human	Pork and/or smoked pork products	[139]
				Insufficiently cooked meat	[119]
		Thailand	Human	Pigs	[140]
		United States	Human	Pork sausage	[141]
		Vietnam	Human	Raw pork	[142]
				Pork	[143]
*Trichinella* T6	Canada, Alaska, Rocky Mountains, and Appalachian Mountains in the United States		Sylvatic carnivores	Carnivores	[33]
		United States		Cougar jerky	[107]
*Trichinella* T9	Japan		Sylvatic carnivores	ND	[33]
		Japan	Human	Bear meat	[144]
*Trichinella* spp.		Argentina	Human	Puma, armadillo and wild boar	[145]
		Austria, Bulgaria, Croatia, Estonia, France, Germany, Greece, Hungary, Italy, Latvia, Lithuania, Netherlands, Poland, Portugal, Romania, Slovakia, Spain, Sweden	Human	Pig and wild boar	[123]
		Bosnia-Herzegovina	Human	Domestic and wild animals	[146]
		Belarus	Human	Domestic and sylvatic mammals	[147]
		China	Human	Pork	[148]
		Czech Republic	Human	–	[149]
		Georgia	Human	Pork	[150]
		Greenland	Human	Polar bear	[151]
		Iran Islamic Rep	Human	Boar meat	[152]
		Ireland	Human	–	[153]
		Israel	Human	Wild pig	[154]
				Wild boar	[155]
		Kazakhstan	Human	Wild boars	[147]
		Kyrgyzstan	Human	–	[149]
		Lao People's Democratic Republic	Human	Raw pork	[156]
		Lithuania	Human	Pork and wild boar	[157]
		Macedonia	Human	–	[149]
		Montenegro, Yugoslavia	Human	Pork	[158]
		Russia	Human	Pork and wild animals (bear, badger, dog)	[159]
				Pork	[160]
		Senegal	Human	Smoked warthog (*Phacochoerus africanus*)	[161]
		Slovakia	Human	Red foxes and wild boars	[99]
		Slovenia	Human	–	[149]
		Switzerland	Human	–	[149]
		China (Taiwan Province)	Human	Soft-shelled turtles	[162]
		Ukraine	Human	Infected game	[163]
		United Kingdom	Human	–	[149]
		Uzbekistan	Human	Wild boar	[147]
Trichuridae, Ransom, 1911					
*Trichuris trichiura* Linnaeus, 1771	Worldwide	Developing regions of Asia, Africa, and Latin America	Human	Ingesting parasitic eggs contaminated in soil, water, vegetables, fruits, etc.	[164, 165]

*ND* None documented

### Dioctophymiasis

The geographical distribution of *D. renale* infection in mammals has been reported in at least 33 countries across Asia, Africa, Europe and South and North America, with Argentina and Brazil each reporting more than 1000 cases. The life cycle involves various paratenic hosts, primarily fish, frogs, and toads. Mustelids and canids predominantly serve as definitive hosts that play a crucial role in sustaining the parasite’s life cycle. At least 49 different mammals have been recognized as definitive hosts for *D. renale*, such as golden jackal, coyote, gray wolf, domestic dog, red wolf, capuchin monkey, fox, wolf, horse, brown rat, brown bear, racoon, mink, and squirrel monkey [166]. The American mink (*Neovison vison*), predominantly found in North America, is identified as the primary definitive host and reservoir, due to the abundant presence of adult parasites of both genders within a single animal host [167].

Dioctophymiasis is a zoonotic disease caused by *Dioctophyme* (or *Dioctophyma*) *renale* (giant kidney worm), and one of the largest known parasitic nematodes infecting humans [168]. This parasite infects humans relatively rarely, and to date, only 10 countries have reported cases of this infection [22] (see Additional file 1: Figure S1). Human infection of *D. renale* usually manifests as nonspecific clinical symptoms including mainly hematuria [22, 169], loin pain [22], nephritis, intermittent kidney pain [169], and renal cyst [170] that may result in worms migrating through the ureter [171]. Sometimes it manifests as weight loss, frequent and urgent urination, fever, anemia, and abdominal pain [22].

### Trichuriasis

Trichuriasis is caused by infection of *Trichuris trichuira*, also known as the human whipworm, and a member of the Trichuridae family. This worm is found worldwide, notably in Asia, Africa and Latin America [164, 165] (see Additional file 1: Figure S2). *T. trichuira* has infected approximately 795 million individuals worldwide [172]. Infection often presents subclinically and moderately, however severe infections, especially among children, can result in weight loss, malnutrition, anemia, and rectal prolapse in addition to watery stools, mucoid mucus, abdominal pain, nausea, and vomiting [173].

### Capillariasis

Capillariasis is caused by *Capillaria*, a genus of the family, Capillaridae, that has 300 species, of which only three are well-known human parasites, including *Capillaria philippinensis* (syn. *Paracapillaria philippinensis*), *Calodium hepaticum* (syn. *Capillaria hepatica*), and *Eucoleus aerophilus* (syn. *Capillaria aerophila*) [12]. Over 300 capillariid species parasitize diverse vertebrate groups worldwide. Identifying them is challenging due to limited taxonomically informative structures, leading to laborious classification and controversial genus definitions that render capillariid taxonomy among the most intricate within Nematoda. An update on a study by Borba [174] focused on examining capillariid eggshell structure via scanning electron microscopy, which compared patterns among 12 species and enhanced taxonomy and species identification. In addition, the study introduced an innovative taxonomic approach by merging biological data with artificial intelligence techniques to characterize capillariid eggs. This method not only aimed to advance current understanding, but also give support for future research, particularly in the taxonomy and diagnosis of both contemporary and ancient capillariids [175].

### Hepatic capillariasis

*Calodium hepaticum* infection causes hepatic capillariasis, which is a zoonotic disease that affects the liver of rodents (main hosts) and various other mammals worldwide [58]. Multiple rodent species that belong to the Muroidea superfamily act as primary hosts for this pathogen. *Calodium hepaticum* has been detected in Muroidean hosts across over 60 countries spanning Africa; Asia; Europe; Oceania; and North, Central and South America. *Rattus norvegicus* (Norway rat) exhibits the highest global prevalence of infection, with rates exceeding 50% reported in various regions across Asia, Europe and South and North America. Other murid species exhibit high prevalence rates in specific regions. For instance, in Asia, *Rattus tanezumi* and *Niviventer fuloscens* (white bellied rat) have been documented with prevalence rates exceeding 50%. Furthermore, *Ondatra zibethicus* serves as a significant host for *C. hepaticum* in North America, while in the UK, *Apodemus sylvaticus* (long-tailed field mice) and *Myodes glareolus* (bank vole) exhibit elevated prevalence rates [176]. While murid rodents act as primary hosts, a wide range of other mammals, including Insectivora (e.g. European hedgehog, shrew), Marsupialia (e.g. opossum, wallaby, kangaroo), Chiroptera (e.g. bat), Artiodactyla (e.g. antelope, peccary, cattle, pig), Lagomorpha (e.g. hare, rabbit, cottontail, pika), Hyracoidea (e.g. hyrax), Perissodactyla (e.g. horse, tapir), Primates (e.g. lemur, monkey, macaque, saki, gorilla, chimpanzee, human), and Carnivora (e.g. skunk, dog, wolf, fox, cat), can also be affected by hepatic capillariasis. In total, over 180 mammalian species, including humans, are recognized as suitable hosts for this parasite [69]. In humans, this worm is responsible for uncommon cases of hepatic capillariosis and spurious infections in patients [177]. It is found throughout the world, and human infection cases of *C. hepaticum* have been documented in different regions of Africa, Asia, Europe, North and South America, and Oceania (see Additional file 1: Figure S3), with 163 individuals affected [58, 59].

Sustained fever, respiratory disorder, abdominal discomfort, diarrhea, leukocytosis, and eosinophilia are common clinical symptoms of *C. hepaticum* infection. Moreover, egg deposition in the liver can result in septal fibrosis and necrotized parasitic granulomas, from which high-intensity infections can induce liver cell disruption and damage [178].

### Intestinal capillariasis

*Capillaria philippinensis* is a pathogen that causes human intestinal capillariasis as well as an emerging zoonotic parasite that has been increasingly evident in the past two decades [12]. Chitwood et al. [179] found the first case of *C. philippinensis* infection in The Philippines, and the parasite became endemic in Thailand in 1973 [180]. Intestinal capillariasis has now spread from endemic areas to several other Asian countries, such as China [72, 85, 86], India [77], Indonesia [78], Japan [79], Republic of Korea [80, 81], and the Lao People’s Democratic Republic [82] (see Additional file 1: Figure S4), and Egypt in Africa [74, 75]. In addition, two cases of infection have been revealed in Europe, one from a Colombian man infected in a nonendemic area, and another from an Italian man who visited an endemic area in Indonesia [73, 78]. More than 2000 residents worldwide have been infected with *C. philippinensis*, resulting in nearly 200 fatalities [62].

Human intestinal capillariasis can present clinically as watery stools, diarrhea, lower limb edema, vomiting, crampy abdominal pain, weight loss, anorexia, and borborygmi [72, 74, 75, 87], which if left untreated can worsen into a serious condition. The severe symptoms of *C. philippinensis,* which include protein-losing enteropathy, hypokalemia (electrolyte loss), and chronic diarrhea [76, 79, 88], can be deadly to humans.

### Pulmonary capillariasis

*Eucoleus aerophilus* is a nematode with a worldwide geographical distribution. It causes lung capillariosis in both wild and domestic animals, including coyote, fox, wolf, cat and dog [181], and sporadic reports have shown its zoonotic potential in humans [182, 183]. The precise life cycle of this parasite, whether direct or indirect, remains unclear, with suggestions that earthworms could serve as potential intermediate or paratenic hosts. The occurrence of *E. aerophilus* has been reported in 36 animal species, encompassing both wild and domestic animals across 38 countries worldwide. The highest prevalence of *E. aerophilus* was detected predominantly in fox, cat and dog, and documented in Lithuania (97.12%), Uruguay (50%) and Italy (19.51%), respectively [184].

The trichuroid parasitic nematode, *E. aerophilus*, is the cause of pulmonary capillariasis. This parasite has been regarded as the most significant respiratory parasite of domestic cat in terms of worldwide distribution over the past three decades, but it rarely infects humans, with only 12 cases being reported in six countries including Russia, France, Iran, Morocco, Serbia and Ukraine [68, 185] (see Additional file 1: Figure S5). Clinical symptoms of *E. aerophilus* infection are coughing, fever, bronchitis, mucoid mucus or blood-tinged sputum, eosinophilia, and dyspnea [183].

### Trichinellosis

Trichinellosis (or trichinosis) is a devastating zoonotic disease caused by members of the genus *Trichinella*, family Trichinellidae, that has a worldwide distribution in domestic and/or sylvatic animals, as well as among humans. An update of this genus in wildlife worldwide was reviewed by Crisóstomo-Jorquera and Landaeta-Aqueveque in 2022 [186]. They found that *Trichinella* spp. had been documented across 129 host species of wild or feral animals in 64 countries. It comprises 13 genotypes, each restricted to specific geographical regions. Europe has the highest number of animals studied, with wild boar being the host species mostly researched. Interestingly, the prevalence of *Trichinella* does not correlate with research efforts, suggesting more focus is needed on animals higher in food chains. Invasive species such as raccoon dog, wild boar, and American mink can serve as important reservoirs. Ultimately, the geographical spread appears to be the primary driver influencing the distribution of *Trichinella* species among hosts. In humans, *Trichinella* infection is related to the consumption of undercooked or raw meat and raw meat derivatives. Each year, 10,000 human incidences of infection from *Trichinella* species are reported from 55 different countries. Nine species of *Trichinella* are suspected currently of infecting humans, including encapsulated (*T. spiralis*,* Trichinella britovi*, *Trichinella murrelli*, *Trichinella nativa*, *Trichinella nelsoni* and two unnamed genotypes: *Trichinella* T6 and T9), and non-encapsulated (*Trichinella papuae* and *Trichinella pseudospiralis*) species [147], with *T. spiralis* being the most pervasive in pig, alongside widespread species of this genus [187]. An update in the literature reported almost 64 countries with human *Trichinella* infection (see Additional file 1: Figure S6), particularly *T. spiralis*, the most frequent species distributed internationally.

*Trichinella britovi* is found widespread in temperate sylvatic carnivores in Europe and Asia. According to reports from Algeria, Bulgaria, France, Greece, Italy, Serbia, Slovakia, Spain, Sweden and Turkey, the majority of *T. britovi* infections in humans have been caused by ingesting raw meat or raw meat products from wild boar and wolf. *Trichinella murrelli* also is present in sylvatic carnivores that dwell in temperate climates in the Nearctic realm. Human infection with *T. murrelli* was first reported in 1985 after feeding infected horses imported into France from Connecticut, resulting in a massive human outbreak [103, 188]. Moreover, sylvatic predators that inhabit frigid regions of Asia, Europe, and North America, including wolf, black and grizzly bear, mountain lion, lynx, fox, and raccoon dog, are known to transmit* Trichinella nativa* [108, 188]. Human infections from *T. nativa* have been confirmed in Canada (northern Ontario and northern Saskatchewan), China (northeast), and the United States between 1996 and 2017 through eating raw meat from bear, dog or mountain lion [104–107]. *Trichinella nelsoni* is found in sylvatic carnivores inhabiting eastern and southern Africa (Kenya, Tanzania, and South Africa), of which spotted hyena is the main reservoir. Less than 100 human infections have been documented with this species in Ethiopia, Kenya, Tanzania, and Senegal spanning the last decade [108]. In addition, *Trichinella* T6 was recognized in carnivores (e.g., fox, bear and walrus) in several Arctic ecosystems in Canada and the United States [33]. Infections in humans have been associated with eating raw game meat, according to 1995 evidence from Idaho in the United States [107]. Sylvatic carnivores in Japan were discovered transmitting *Trichinella* T9. The intake of raw bear meat correlated with *Trichinella* T9 infection, causing an outbreak of trichinellosis in 2016, which affected 21 individuals across Japan [144]. *Trichinella papuae* was discovered by Pozio et al. in 1999, and is the most recent species infecting sylvatic swine in Papua New Guinea [189]. Mammals and reptiles are the principal reservoir hosts for this species [33]. It is interesting to note that during outbreaks from 2006 to 2020, human infections for this species have been reported in Thailand [111, 112], Malaysia [110], and Cambodia [109]. In Tasmania, *Trichinella pseudospiralis* has been detected in both mammals and birds [147]. Human infections from eating domestic or sylvan animals have been revealed in Tasmania, Thailand, France, Italy, and Kamchatka Krai. Besides, this parasite was the causative agent of the 2015 trichinellosis epidemic in northern Italy [114].

Patients in the gastrointestinal phase of *Trichinella* spp. infection are generally asymptomatic or present mild clinical manifestations, such as nausea, diarrhea, vomiting and abdominal pain after 1–2 days, as a result of the first stage larvae penetrating the small intestine [190]. Skeletal muscles, periorbital edema, fevers, myalgias, myositis, encephalitis, myocarditis, high levels of eosinophils, white blood cells, and muscle enzymes are among the classic clinical symptoms of the subsequent systemic and muscular phase, which is brought on by larvae entering the lymphatic circulation and blood vessels [161, 190]. Clinical symptoms might range from moderate to severe, depending on the number of parasitic worms taken from meat. Severe infections, however, ultimately result in death [117, 190].

### Parasitic myositis

Parasitic myositis is caused by the muspiceoid nematode, *Haycocknema perplexum*, family Robertdollfusidae, and is suspected of being a zoonotic disease. It was reported initially in 1998 and isolated from a man in Tasmania, Australia [65]. Human myositis has now spread from endemic areas in Australia, including tropical north Queensland and Tasmania [21, 90] (see Additional file 1: Figure S7), and its global distribution remains unclear. This worm is a rare cause of myositis in humans, with only 9 patients being publicized in Australia between 1998 and 2016 [90]. Human infection with *H. perplexum* generates mild eosinophilia, elevated creatine kinase, muscular weakness, dysphagia, and weight loss [24, 90, 191].

### Disease prevention and control

While the majority of human parasites in the Adenophorea class are zoonotic and propagate through the ingestion of undercooked meats by humans, some species (*T. trichiura* and *E. aerophilus*) infect by consumption of embryonated eggs from soil. However, due to the unclear life cycle of *H. perplexum,* there are no specific recommendations for its prevention and control. Since no vaccines are available, prevention relies on education, hygiene measures, and managing animal hosts (Table 2).

**Table 2:** Summary of major prevention and control measures for human parasitic diseases of the Adenophorea class of nematodes

**Measure**	**Details**
Education and hygiene	- Educate consumers on the risks of eating raw/undercooked meat from domestic/wild animals (fish, frog, pig, etc.). - Promote hand washing with soap before eating.
Food safety	- Cook meat at no less than 71 °C (160 °F). - Freeze meat at no less than -15 °C (5 °F) (not recommended for wild game due to freeze-resistant parasites). - Use irradiation (0.3 kGy).
Sanitation (WASH Program)	- Improve water quality and quantity. - Increase access to latrines. - Manage fecal waste. - Promote personal hygiene.
Preventive chemotherapy	- Administer single doses of oral mebendazole or albendazole. - Target children, women of reproductive age, and at-risk populations. - Aim for at least 75% coverage.
Animal control	- Regular deworming of animals. - Prevent animals from accessing contaminated environments. - Regular testing/treatment for parasitic infections. - Rodent control.
Research and development	- Vaccines. - Probiotics (e.g., *Lactobacillus* to prevent *T. spiralis* and *T. britovi*). - Chemicals such as OX02983 to inhibit egg development and hatching.

*WASH* Water, sanitation, and hygiene

In minimizing parasitic infection, consumers should be informed about the risks of eating raw or undercooked meat from domestic and wild animal carriers of parasites (such as *D. renale*, *Capillarea* and *Trichinella*) [22, 33, 192]. The International Commission on Trichinellosis advises using test-and-slaughter techniques, cooking, or irradiating to prevent infection [193]. However, freezing is ineffective for wild game, due to freeze-resistant *Trichinella* taxa such as *T. britovi* and *T. nativa* [194]. Furthermore, the Global Water, Sanitation, and Hygiene (WASH) program, guided by the World Health Organization (WHO), helps in reducing trichuriasis and other soil-transmitted helminth infections by improving water quality and quantity, access to latrines, fecal waste management, and personal sanitation and hygiene. Preventive chemotherapy significantly reduces the prevalence and impact of soil-transmitted helminthiases, with an up to 80% decrease in parasite burden and prevalence in endemic areas [195–197]. In 2017, the WHO revised guidelines for preventive chemotherapy against soil-transmitted helminth infections (STHs) in endemic regions [198]. These programs, which administer single doses of mebendazole or albendazole to at-risk groups, aim to reduce trichuriasis prevalence [199]. By 2017, 598 million children (69% of the at-risk population) were treated, nearing the 2020 target of 75% coverage [200]. However, reinfection in areas with poor sanitation and hygiene remains a problem [201], emphasizing the need for better WASH programs to control STH infections [200].

Animal control measures reduce animal-to-human transmission through deworming regularly, preventing access to contaminated environments, and treating infected animals. Regular testing and treatment can eliminate fecal contamination in animals reared in uncontaminated areas [202, 203]. Treating infected cats with parasiticide (1% spot-on moxidectin) effectively reduces *Capillaria* spp. eggs [204]. Rodent control and appropriate sanitation help to prevent the transmission of *C. hepaticum*, *Trichinella*, and *D. renale*. In addition to current measures, other prevention methods are being researched, such as vaccines [205] and probiotics to prevent *T. spiralis* [206] and *T. britovi* [207] infections. The chemical, dihydrobenzoxazepinone OX02983, can inhibit egg development and hatching by breaking the life cycle of *T. trichiura* [208]. These innovative techniques have the potential for employment as efficient prevention and control measures.

## Conclusions

Human infection by ANs remains an important worldwide health concern. Approximately 15 nematode species from diverse tropical and subtropical regions have been linked to human infections. Dioctophymiasis, trichuriasis, capillariasis, trichinellosis, and myositis are human parasitic diseases caused by ANs, each with a distinct geographical distribution. These diseases pose significant threats to human health, due to their multifaceted effects on various organs, which often lead to severe complications and even mortality.

Humans serve as definitive and accidental hosts of ANs, and are infected by ingesting undercooked or raw meat and food contaminated with parasitic eggs. To interrupt the cycle of disease transmission, significant progress has been made in preventive and control measures, primarily focusing on animal control. Additionally, efforts have been made to emphasize the importance of education, sanitation, and hygiene practices to mitigate the risk of contracting these infections.

Based on epidemiological data and studies of the parasitic life cycle, the updated information in this review supports the prevention and control of parasitic infections, as well as further surveillance and forecasting of human-parasite infection outbreaks in both endemic and non-endemic areas. However, the life cycle and particular host of *H. perplexum* are still unknown, hence, no recommendations for avoiding and managing them are currently available. Comprehensive molecular epidemiology research should be a component of further studies to better understand of the distribution, parasitic ecology and parasitic infections in animals and humans, potentially leading to the establishment of new strategies for monitoring, predicting, preventing, and actively managing parasitic diseases caused by ANs.

### Supplementary Information

Supplementary Material 1.

## Data Availability

The datasets used and/or analyzed during this study are available from the corresponding author upon reasonable request.

## Abbreviations

ANs: Adenophorean nematodes
Ch: *Calodium hepaticum*
Cp: *Capillaria philippinensis*
Dr: *Dioctophyme renale*
Ea: *Eucoleus aerophilus*
Hp: *Haycocknema* *perplexum*
L_1_: First-stage larvae
L_3_: Third-stage larvae
Tr: *Trichinella* spp.
Tt: *Trichuris trichuira*
