# Supplementary material for: Human parasitic infections of the class Adenophorea: global epidemiology, pathogenesis, prevention and control
Source: Infect Dis Poverty. 2024 Jun 20;13:48. doi: 10.1186/s40249-024-01216-1 (PMC11188577; doi:10.1186/s40249-024-01216-1)
Supplement: Supplementary file 1 — Supplementary Material 1. [file 40249_2024_1216_MOESM1_ESM.docx]

Supplementary Table S1 – Search strategy

| **Databases** | **Search strategy** |
| --- | --- |
| Pubmed | (Nematode[tiab] OR “nematode humans”[tiab] OR (nematode[tiab] AND humans[tiab]) OR “nematode infection”[tiab] OR “Adenophorea”[tiab] OR “Adenophorea human infection”[tiab] OR “Adenophorea morphological”[tiab] OR “Adenophorea pathogenesis”[tiab] OR “nematode morphology”[tiab] OR “global epidemiology of nematodes”[tiab] OR “nematode pathology in humans”[tiab] OR “nematode life cycle”[tiab] OR "dioctophyme renale"[All Fields] OR "dioctophymiasis" OR "haycocknema perplexum"[tiab] OR "parasitic myositis" OR “calodium hepaticum”[tiab] OR “hepatic capillariasis” OR “eucoleus aerophilus” [tiab] OR “pulmonary capillariasis” OR “capillaria aerophila”[tiab] “capillaria philippinensis”[tiab] OR “intestinal capillariasis” OR “capillariasis” OR “trichuris trichiura”[tiab] OR “trichuriasis” [tiab] OR “trichinella”[tiab] OR “trichinellosis”[tiab] OR “human cases” OR “human parasites” OR “parasite infections” OR “parasite prevalence” OR “prevention” AND “control”) |
| Embase | (Nematode:ab,ti OR “nematode humans”:ab,ti OR (nematode:ab,ti AND humans:ab,ti) OR “nematode infection”:ab,ti OR Adenophorea:ab,ti OR " Adenophorea human infection":ab,ti Adenophorea morphological:ab,ti OR " Adenophorea pathogenesis":ab,ti OR nematode morphology:ab,ti OR "global epidemiology of nematodes":ab,ti OR "nematode pathology in humans":ab,ti OR “nematode pathology in humans”:ab,ti OR "dioctophyme renale":ab,ti OR "dioctophymiasis":ab,ti OR “haycocknema perplexum”:ab,ti OR “parasitic myositis”:ab,ti OR “calodium hepaticum”:ab,ti OR "hepatic capillariasis":ab,ti OR “eucoleus aerophilus”:ab,ti OR "pulmonary capillariasis":ab,ti OR "capillaria aerophila":ab,ti OR "capillaria philippinensis":ab,ti OR "intestinal capillariasis":ab,ti OR "capillariasis":ab,ti OR "trichuris trichiura":ab,ti OR " trichuriasis":ab,ti OR "trichinella":ab,ti OR "trichinellosis":ab,ti OR "human cases" OR "human parasites" OR “parasite infections” OR “parasite prevalence” OR “prevention” AND “control”) |

**The data visualization for human parasitic infections of the Class Adenophorea: global epidemiology, pathogenesis, prevention and control**

**Method:**

**Data visualization**

Geological data for this study were obtained from Natural Earth Data, a public domain map dataset known for its detailed features. The study utilized the ‘50m Cultural Vectors’ and ‘50m Physical Vectors’ datasets, which offer comprehensive global coverage at a 1:50 million scale using the Equal Earth Projection (units: meters). These datasets were downloaded from the Natural Earth Data website (https://www.naturalearthdata.com/downloads/). To combine this geological data with epidemic information, Python libraries such as GeoPandas, geo_northarrow, and Matplotlib were used for processing and visualization.

**Results:**

**Supplementary Figure S1 Global distribution of human dioctophymiasis cases.**

**
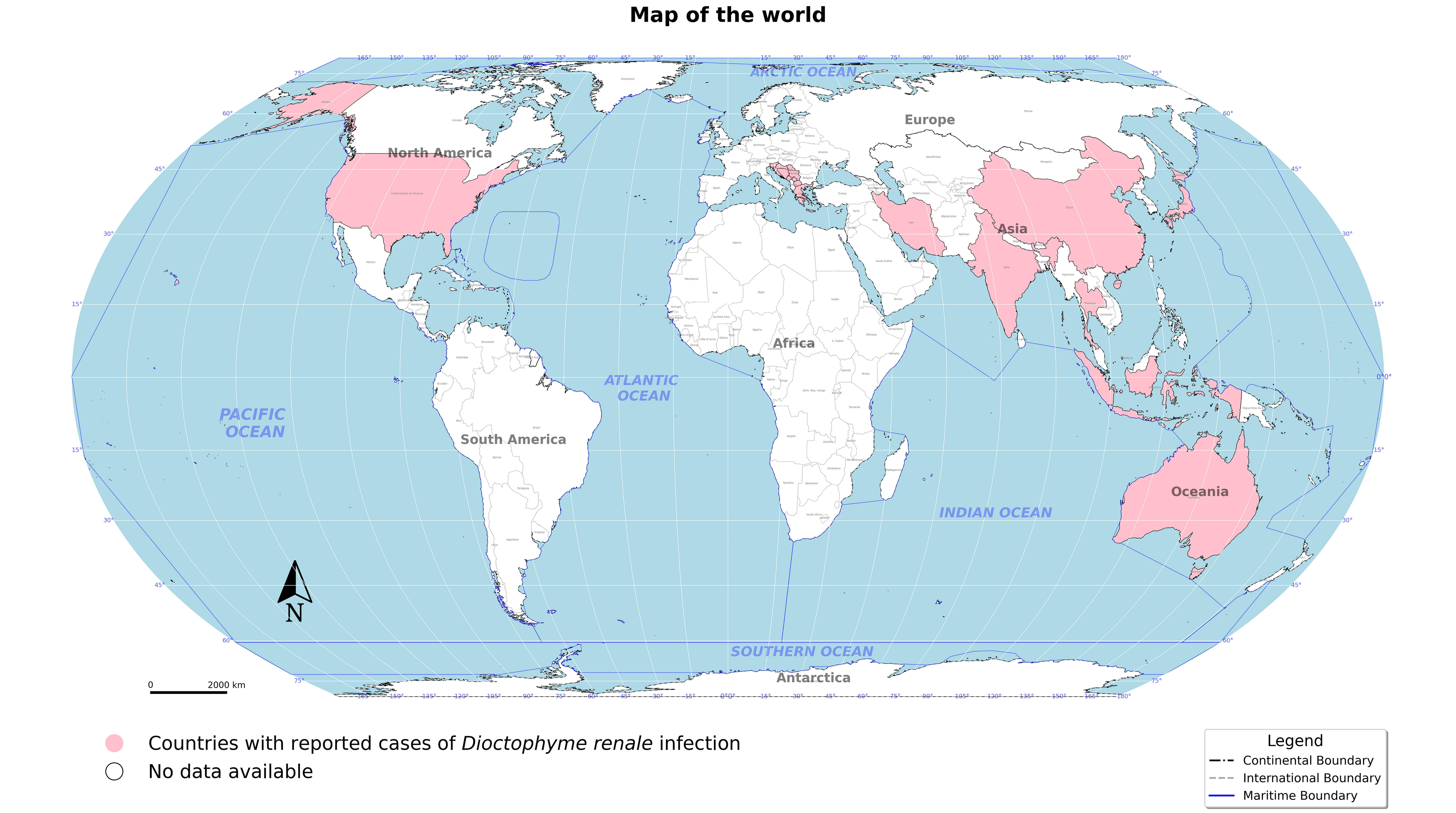
**

**Supplementary Figure S2 Global distribution of human trichuriasis cases.**

**
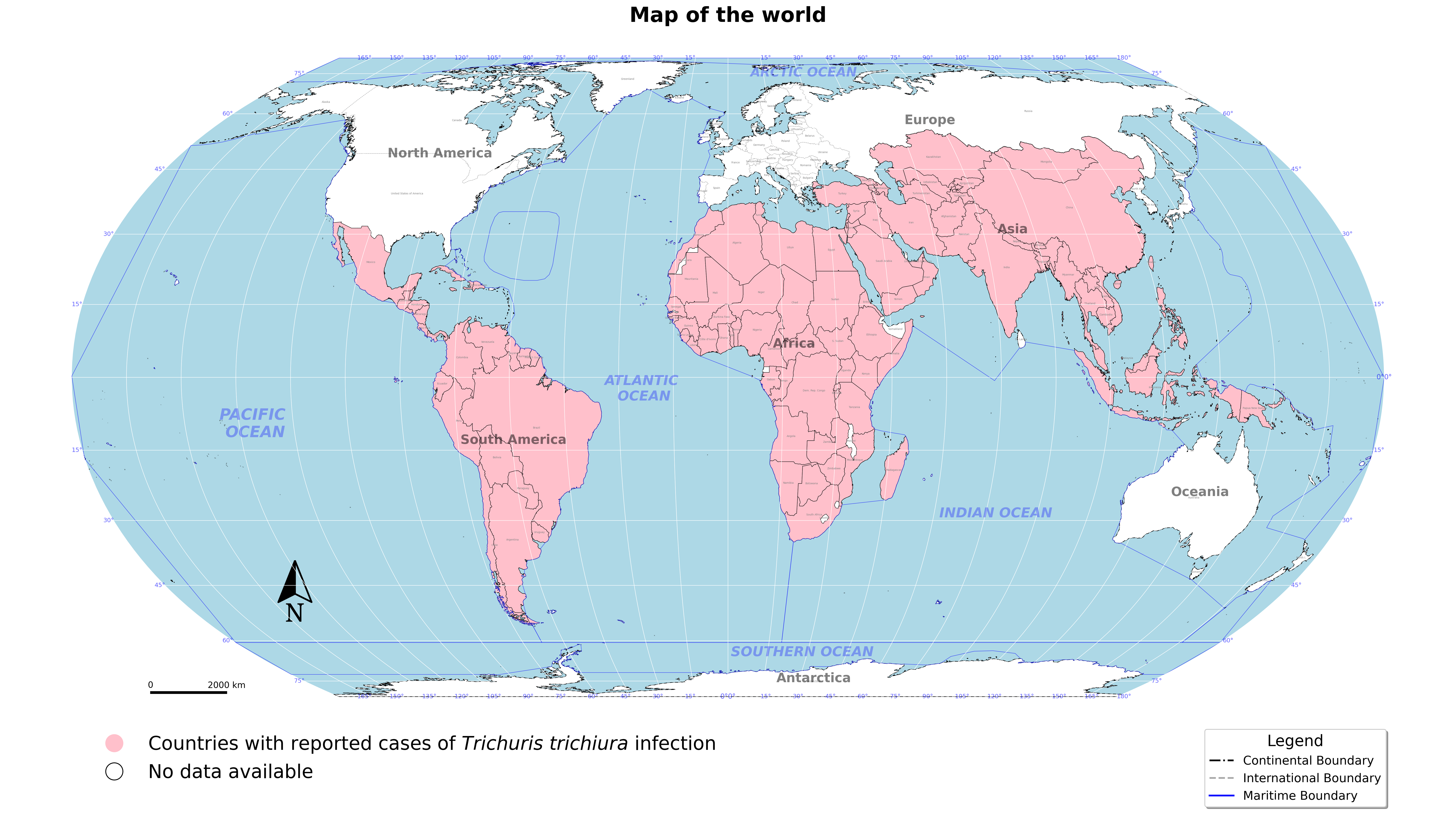
**

**Supplementary Figure S3 Global distribution of human hepatic capillariasis cases.**

**
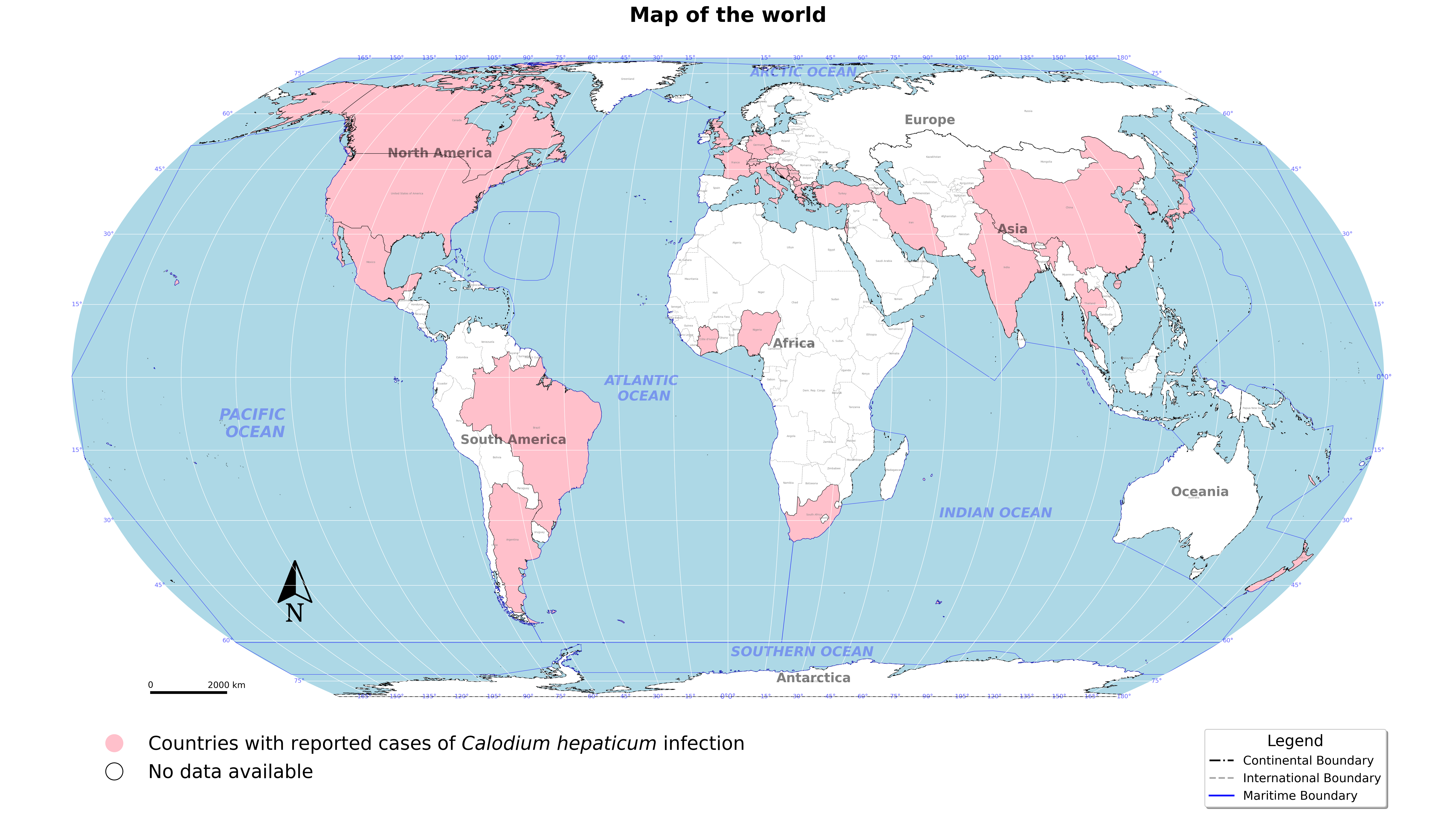
**

**Supplementary Figure S4 Global distribution of human intestinal capillariasis cases.**

**
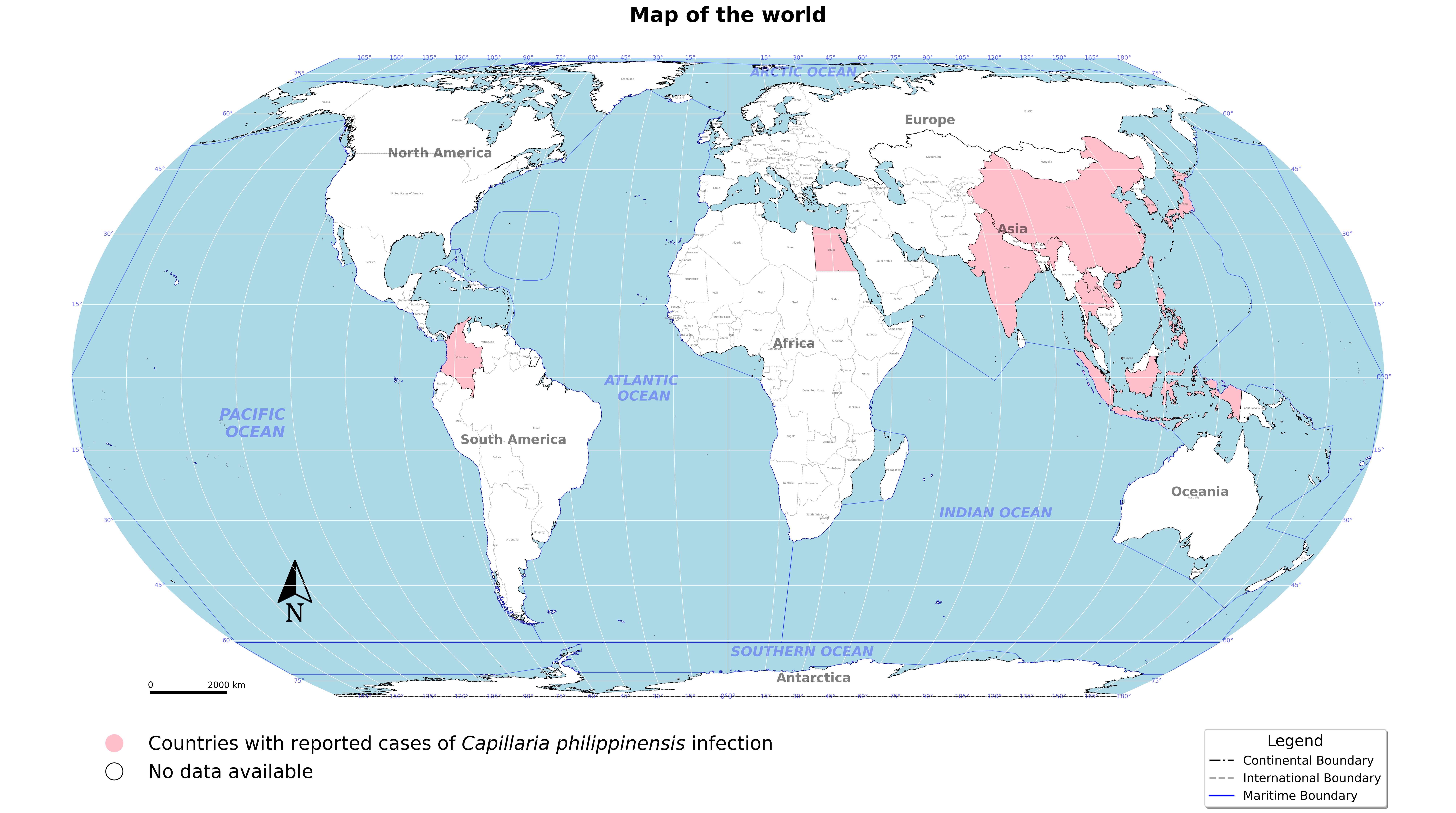
**

**Supplementary Figure S5 Global distribution of human pulmonary capillariasis cases.**

**
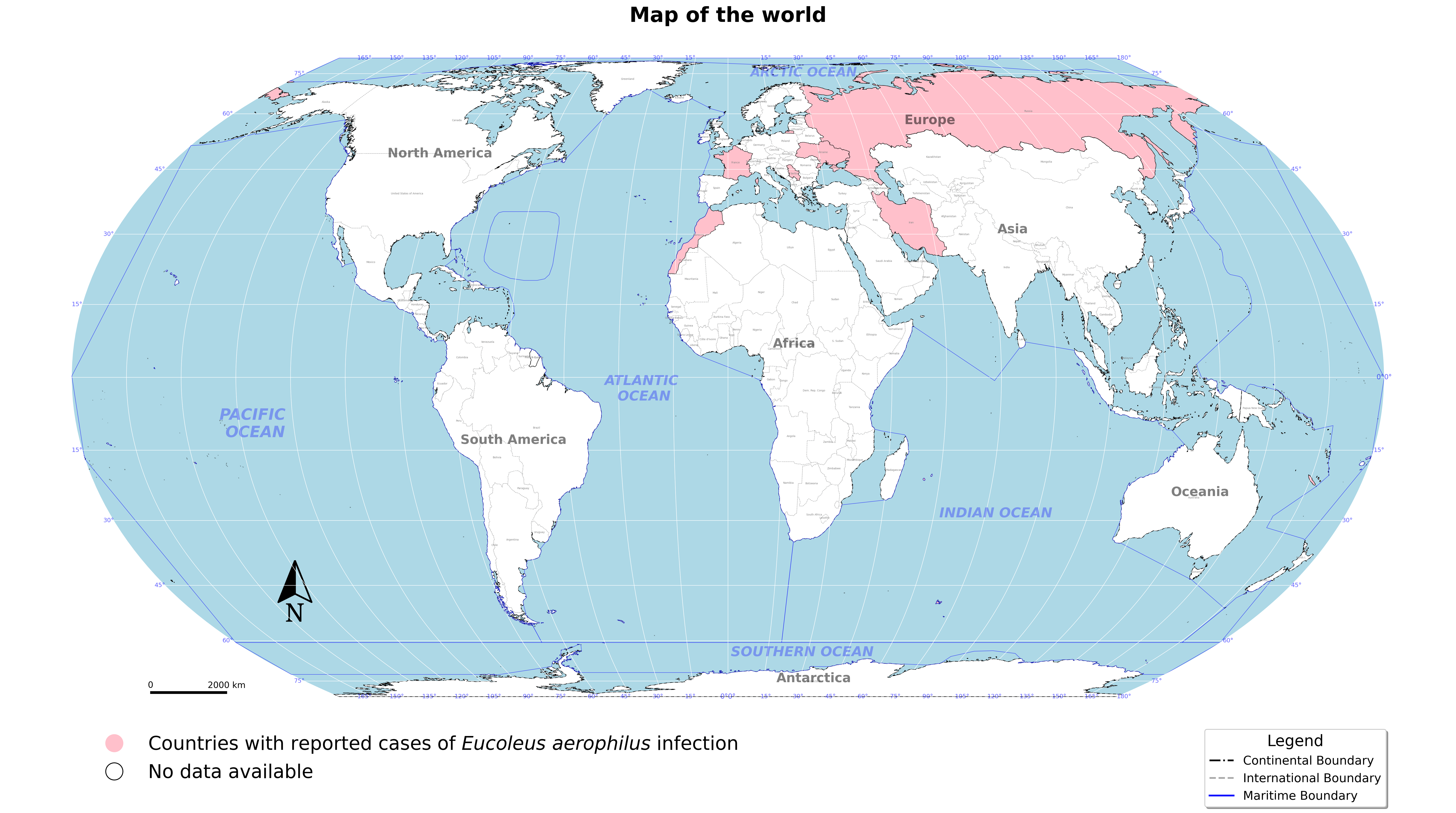
**

**Supplementary Figure S6 Global distribution of human trichinellosis cases.**

**
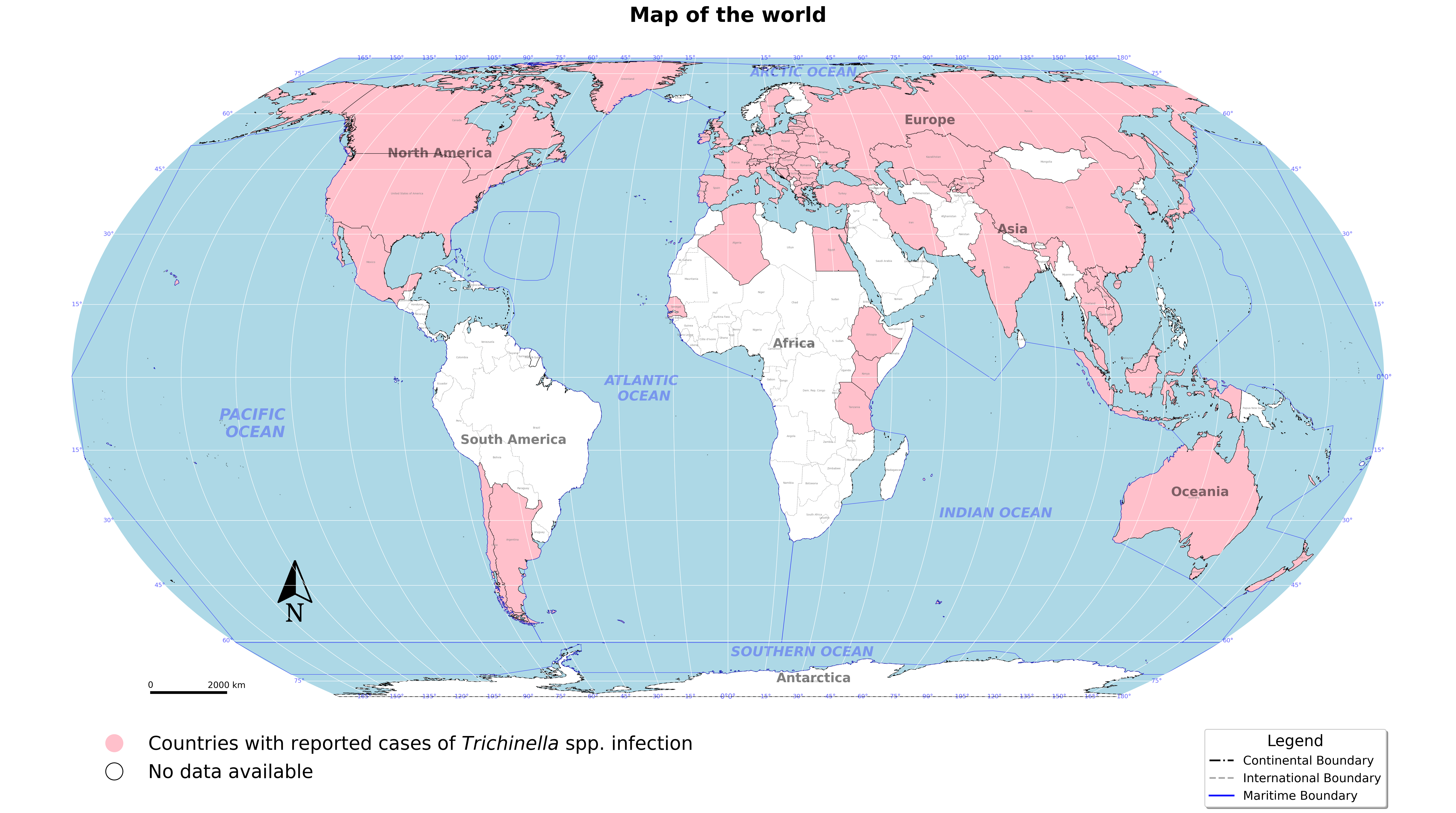
**

**Supplementary Figure S7 Global distribution of human myositis cases.**

**
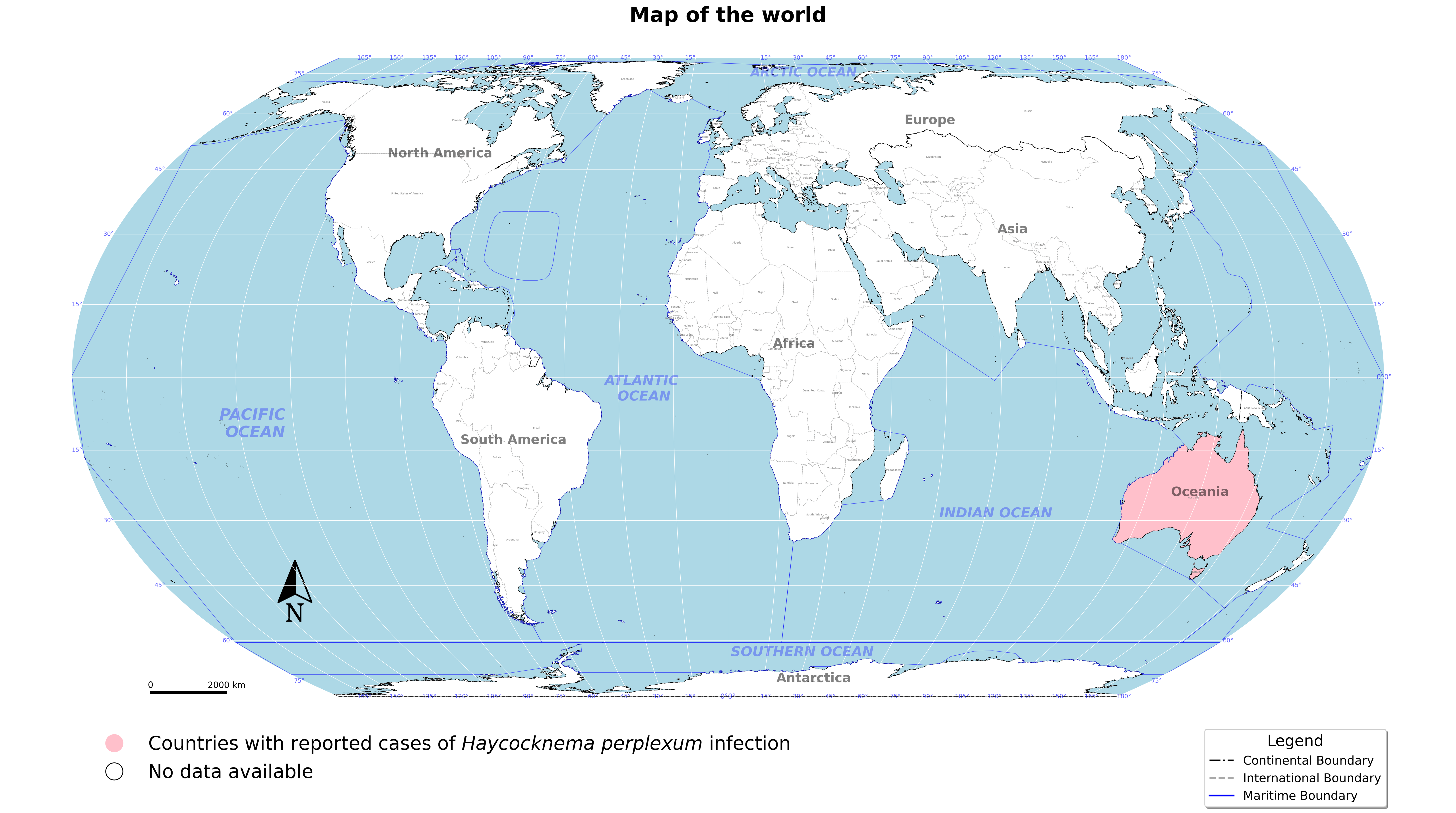
**
